# Optimization of key factors affecting hydrogen production from sugarcane bagasse by a thermophilic anaerobic pure culture

**DOI:** 10.1186/s13068-014-0119-5

**Published:** 2014-08-20

**Authors:** Zhicheng Lai, Muzi Zhu, Xiaofeng Yang, Jufang Wang, Shuang Li

**Affiliations:** Guangdong Key Laboratory of Fermentation and Enzyme Engineering, School of Bioscience and Bioengineering, South China University of Technology, Panyu District, Guangzhou, 510006 China

**Keywords:** Biohydrogen, *Thermoanaerobacterium aotearoense* SCUT27/Δ*ldh*, Non-sterilization, Sugarcane bagasse, Acid hydrolysate, Dark fermentation

## Abstract

**Background:**

Hydrogen is regarded as an attractive future energy carrier for its high energy content and zero CO_2_ emission. Currently, the majority of hydrogen is generated from fossil fuels. However, from an environmental perspective, sustainable hydrogen production from low-cost lignocellulosic biomass should be considered. Thermophilic hydrogen production is attractive, since it can potentially convert a variety of biomass-based substrates into hydrogen at high yields.

**Results:**

Sugarcane bagasse (SCB) was used as the substrate for hydrogen production by *Thermoanaerobacterium aotearoense* SCUT27/Δ*ldh*. The key parameters of acid hydrolysis were studied through the response surface methodology. The hydrogen production was maximized under the conditions of 2.3% of H_2_SO_4_ for 114.2 min at 115°C. Using these conditions, a best hydrogen yield of 1.86 mol H_2_/mol total sugar and a hydrogen production rate (HPR) of 0.52 L/L · h were obtained from 2 L SCB hydrolysates in a 5-L fermentor, showing a superior performance to the results reported in the literature. Additionally, no obvious carbon catabolite repression (CCR) was observed during the fermentation using the multi-sugars as substrates.

**Conclusions:**

Considering these advantages and theimpressive HPR, the potential of hydrogen production using *T. aotearoense* SCUT27/Δ*ldh* is intriguing. Thermophilic, anaerobic fermentation using SCB hydrolysates as the medium by this strain would be a practical and eco-friendly process.

**Electronic supplementary material:**

The online version of this article (doi:10.1186/s13068-014-0119-5) contains supplementary material, which is available to authorized users.

## Background

The depletion of fossil fuels has triggered concerns over the development of renewable energy sources. Although there are still some difficulties in hydrogen commercialization, such as high production costs, technical storage, and distribution [1], biohydrogen production is exhibiting perhaps the greatest potential as an alternative to fossil fuels [2] because of its clean, high energy content per unit of weight (142 KJ/g) and zero greenhouse gas emissions generated by oxidative combustion. Currently, most commercial hydrogen is obtained from steam reforming of hydrocarbons. High temperature electrolysis of alkaline solutions has been extensively developed in recent years, accounting for 4% of the current total hydrogen production [3]. However, all these processes are highly energy consuming and require high temperatures (>850°C) [4], and thus are not sustainable. Biological methods are attractive because of their low energy requirements compared with those of chemical processes. The promising processes of biohydrogen production include light fermentation by photosynthetic bacteria and algae and dark fermentation by strictly or facultatively anaerobic bacteria. Since large amounts of lignocellulosic waste are made every year on earth [5], dark fermentation is a key technology for the production of hydrogen from agro-industrial by-products [1]. Various types of microorganisms can play a role in hydrogen formation by dark fermentation. However, thermophiles are energetically more favorable for hydrogen production, generating higher H_2_ yields and fewer undesirable by-products than mesophiles [6]. Moreover, strictly anaerobic thermophilic conditions seem to restrict contamination by other microorganisms [7].

Lignocellulosic biomass contains three main components: cellulose, hemicellulose, and lignin. Cellulose and hemicellulose are polysaccharides composed of sugar molecules, which could be used as a substrate for hydrogen production through dark fermentation. Sugarcane bagasse (hereafter SCB) offers numerous advantages with respect to its low ash content compared with other crop residues, such as rice straw and wheat straw, when used for bioprocessing purposes. Moreover, SCB is a richer solar energy carrier due to its higher yields in mass per unit area of cultivation and its annual regeneration capacity [8]. However, the lignin fractions in SCB form a formidable barrier to microbial digestion during fermentation. SCB pretreatment has been found useful in easing the difficulties of microorganisms’ attack by enlarging the inner surface area of substrate particles. The pretreatment technology also fractionates SCB and results in partial solubilization and degradation of cellulose and hemicellulose [9]. Previous studies have reported on SCB pretreatment using either physical or chemical methods, such as acid [10-12], alkali [13], and steam [14-16]. However, it has been generally agreed that acid pretreatment is the method of choice in several model processes [17]. One of the most cost-effective pretreatments is to use dilute acid at moderate temperatures. Despite the fact that lignin cannot be removed by this process, its splitting renders a significant improvement in sugar yield compared to other processes.

After the pretreatment of SCB, the released fractions containing cellulose and hemicellulose must be converted to glucose and other monomeric sugars, which can be achieved by acid hydrolysis. Although high sugar recovery efficiency can be achieved through concentrated acid hydrolysis, problems associated with equipment corrosion and higher energy demand areunavoidable challenges. Also, dilute acid hydrolyzation consumes acid in small amounts, which implies that it is more friendly to the environment.

In our previous work, a new strain, *Thermoanaerobacterium aotearoense* SCUT27/Δ*ldh*, was isolated and engineered which can generate a much higher hydrogen yield than most strains reported in the literature [18]. In this study, we used SCB hydrolysate to produce hydrogen with the SCUT27/Δ*ldh* strain. Our preliminary study indicated that the SCUT27/Δ*ldh* could utilize xylan and dextran as the sole carbon source to grow and release hydrogen without any enzyme addition. Furthermore, a related strain (LA1002) [19] could produce lactic acid efficiently under non-sterilized conditions without contamination. These facts encouraged us to explore hydrogen production with this strain using dilute acid-hydrolyzed SCB as the substrate, without sterilization. Herein, we have aimed to optimize the conditions for SCB hydrolysis to achieve more hydrogen with dilute sulfuric acid at relatively moderate temperatures through the use of the response surface methodology. The optimum conditions obtained were further confirmed in a larger batching process to produce hydrogen in a 5-L fermentor containing 2 L hydrolysate.

## Results and discussion

### Influence of carbon source on hydrogen production

Prior to the utilization of sugarcane bagasse (SCB) hydrolysate for hydrogen production, a set of experiments was carried out in 125-mL serum bottles with a working volume of 50 mL. The fermentations were performed using a modified MTC medium [18] supplemented with different sugars or sugar mixtures as the carbon source to determine the cell growth and hydrogen production of *T. aotearoense* SCUT27/Δ*ldh*. The concentrations of sugars were at the same levels of 10 g/L. In the batch tests, the cell density and the produced hydrogen were determined, and all the experiments were carried out in triplicate.

**Figure 1:**
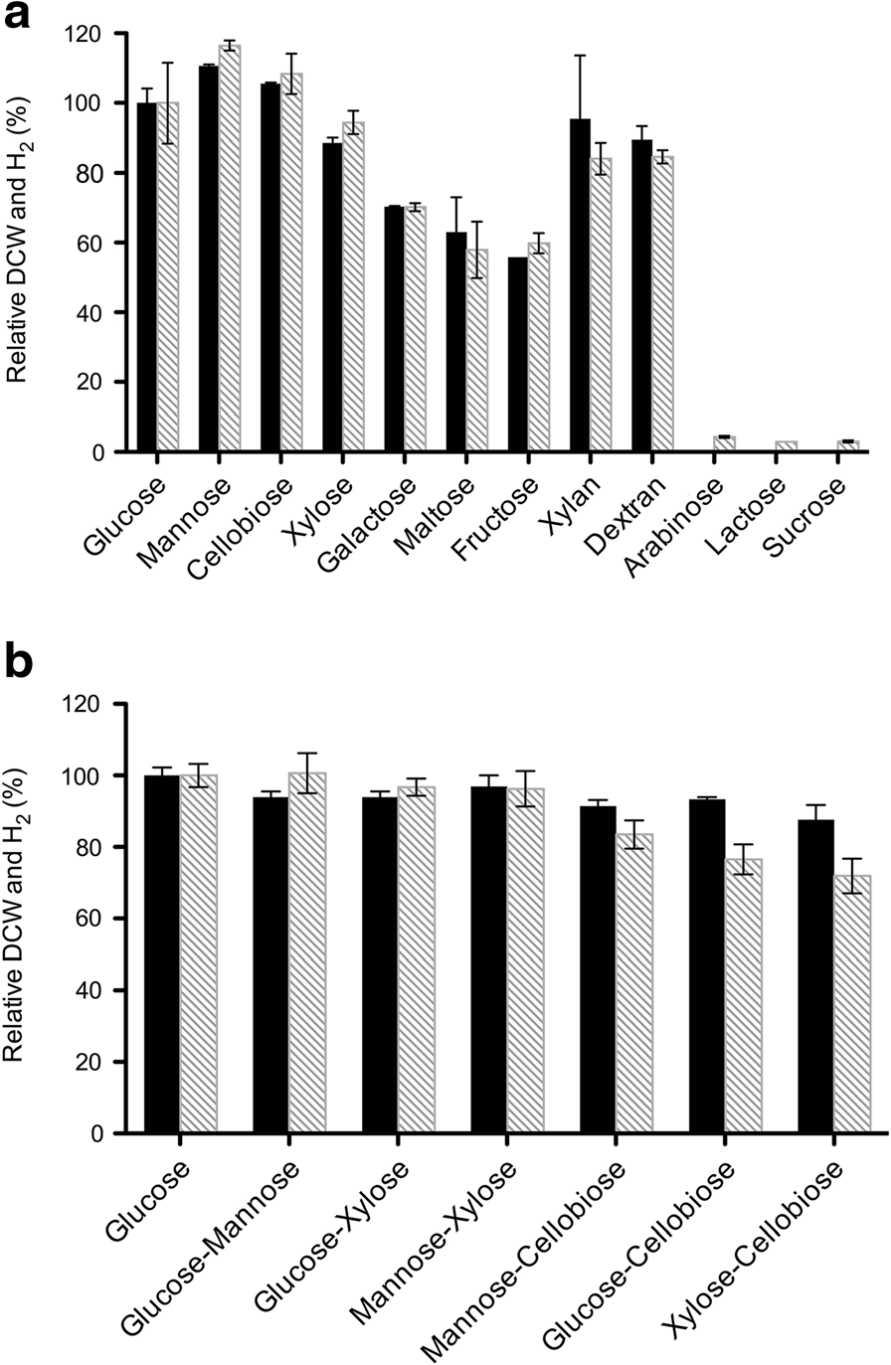
**Comparison of relative DCWs (black bars) and hydrogen production (gray-shaded bars) for** ***T. aotearoense*** **SCUT27/**Δ***ldh*** **using different sugars as carbon source. (a)** Using single sugar as carbon source, **(b)** using sugar mixture as carbon source (1:1, *w*:*w*). Relative DCW and hydrogen production were calculated with respect to that using glucose as the sole carbon source. The error bars represent the standard deviation (SD) (n = 3). The data were collected and calculated after 24 h incubation at 55°C, except those from beechwood xylan and dextran, which were recorded after 48 h cultivation. Experiments were carried out in triplicate.

Using the results from glucose as a control, the relative dry cell weight (DCW) and hydrogen productivities were determined and are presented in Figure 1. In addition to glucose, SCUT27/Δ*ldh* readily degraded xylose, mannose, cellobiose, fructose, galactose, maltose, beechwood xylan, and dextran to grow and produce hydrogen (Figure 1a). However, microorganisms could not efficiently grow using arabinose, lactose, and sucrose as the sole carbon source. In terms of a strong correlation between cell growth and hydrogen release [20], little hydrogen was detected using these sugars as the substrate with this strain. Among the different carbon sources examined, mannose achieved the highest hydrogen production, followed by cellobiose as a single carbon source. The final amount of hydrogen in the mixture was not distinctively different from that in the single sugar medium (Figure 1b). In addition, glucose, mannose, cellobiose, and xylose in a single sugar medium or in the mixed sugar medium were completely consumed after 24 h fermentation.

Generally, the fermentation of pentose (xylose, arabinose) is difficult for an efficient production of biofuels from lignocellulosic materials, because only a limited number of microorganisms can utilize pentose and other monosaccharides released from hemicelluloses (mannose, galactose) into bioproducts with a satisfactory yield and productivity. Although SCUT27/Δ*ldh* cannot utilize arabinose very effectively, it can convert xylose, mannose, and galactose to hydrogen efficiently, with more than 70% relative hydrogen productivity compared to that using glucose as the sole carbon source (Figure 1a). It is worth noting that SCUT27/Δ*ldh* has a strong capability to utilize beechwood xylan and dextran as a single carbon source to support cell growth and hydrogen release without the addition of any cellulase or xylanase, because of its high level of cellulase and xylanase expression (unpublished data). In general practice, enzymatic hydrolysis is required for lignocellulosic biomass utilization in biofuel fermentation to obtain simple reducing sugars or monosaccharides [21]. The sugar utilization by the engineered strain of SCUT27/Δ*ldh* is considered very valuable for biohydrogen production using natural lignocellulosic materials as the feedstock.

### Effects of inhibitors on cell growth

During the dilute acid pretreatment of SCB, many toxic compounds are produced or introduced which have potentially inhibitory effects on cell growth, thus posing a serious challenge for the feasibility of lignocellulosic biofuel production [22]. An understanding of the inhibitors’ effects on *T. aotearoense* cell growth could help us to determine the further processing for hydrogen production after SCB hydrolysis.

**Figure 2:**
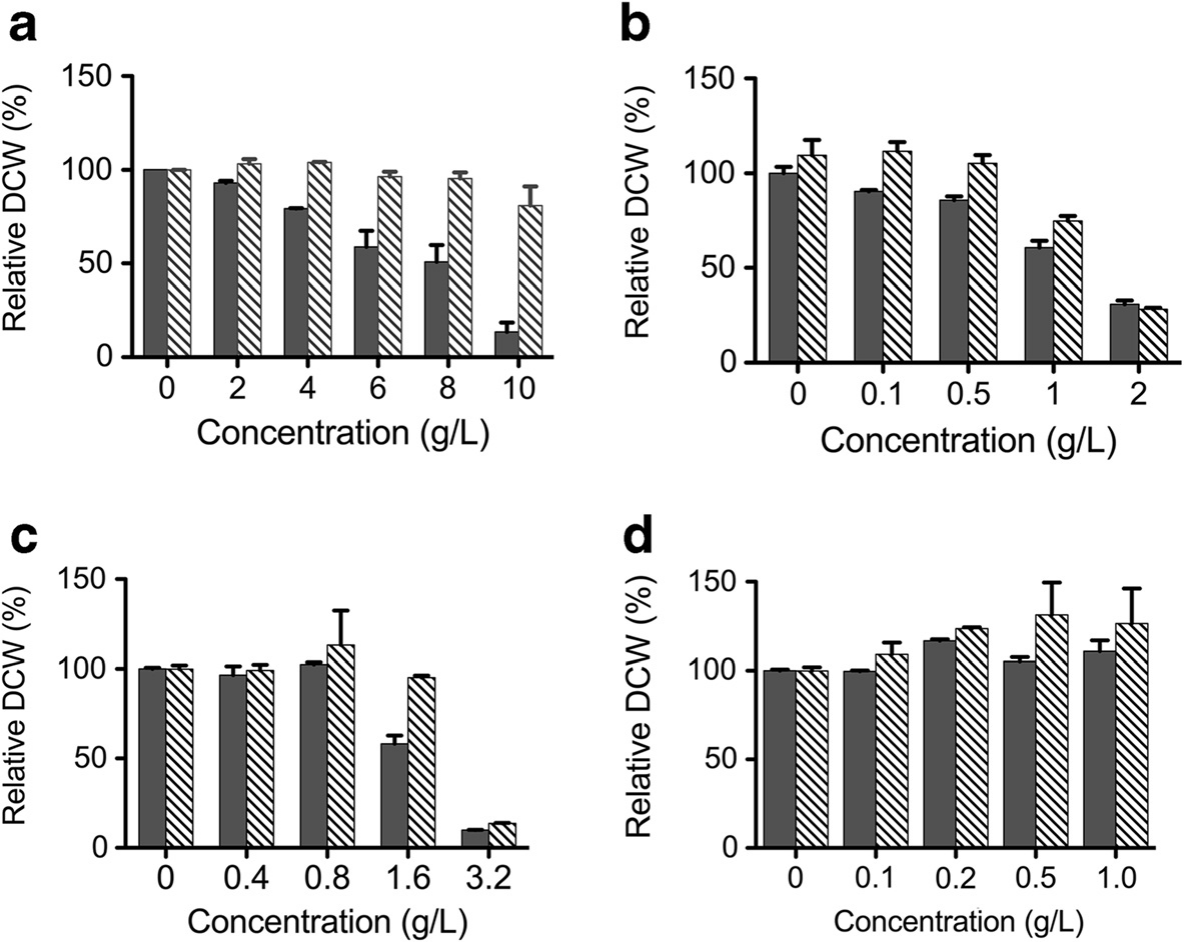
**Cell growth of** ***T. aotearoense*** **SCUT27/Δ** ***ldh*** **after different incubation time at 55°C in the modified MTC medium supplemented with different concentrations of toxic agents.** Fermentation time of 12 h is showed as black bars and 24 h is presented as gray-shaded bars. **(a)** acetic acids, **(b)** phenols **(c)** furfurals and **(d)** 5- hydroxymethyl furfural. Experiments were performed in triplicate.

Figure 2a shows the final cell density in 125-mL serum bottles at 55°C for 12 or 24 h, supplemented with different concentrations of inhibitors. The final DCW of SCUT27/Δ*ldh* decreased as the acetic acid concentration increased over the range of 0 to 10 g/L, with a reduction of 90% at 10 g/L acetic acid. However, the differences in the final cell mass under different acetic acid concentrations were narrowed when the cells were cultured at 55°C for 24 h. This indicated that a high concentration of acetic acid could result in an extended lag phase. There was no obvious suppression of cell growth in concentrations of 0 to 1 g/L for phenol and 0 to 1.6 g/L for 2-furaldehyde (furfural), respectively (Figure 2b and c). However, the inhibition phenomenon became apparent at concentrations higher than 2 g/L (phenol) and 3.2 g/L (furfural). Experimental results showed that the inhibitory effect was not relieved by extending the incubation time to 24 h. Furthermore, there was no substantial distinction among the final cell densities after 12 h or 24 h fermentation in the observed concentrations of 5-hydroxymethyl furfural (HMF) (Figure 2d). Actually, the maximum concentrations of furfural and HMF produced from the dilute acid hydrolysis of SCB in this study were lower than 0.8 and 0.2 g/L, respectively. Thus, no further investigation was applied to study the effects of these two inhibitors on the hydrogen production by SCUT27/Δ*ldh*.

### Acid hydrolysis of SCB

**Figure 3:**
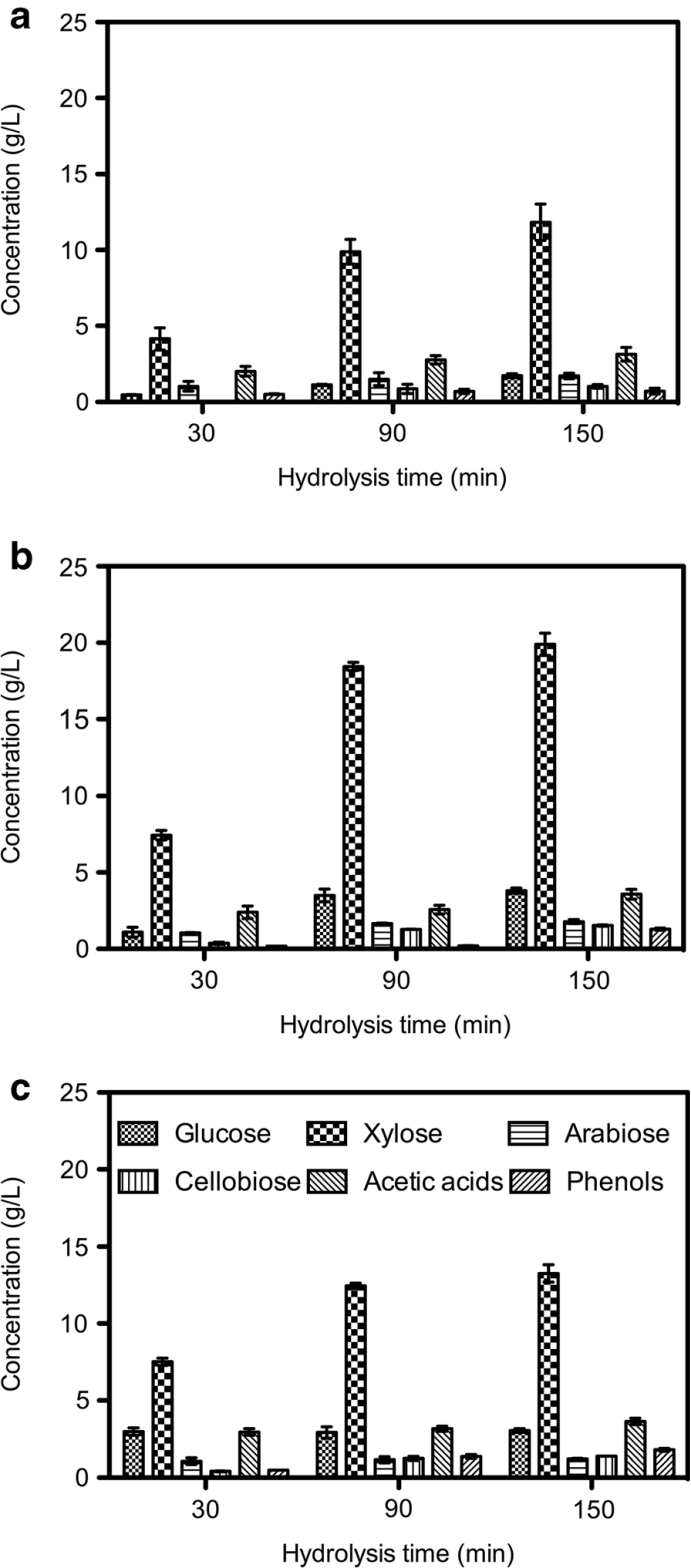
**Sugars and inhibitors released from SCB at different hydrolysis times using different concentration of H**_**2**_ **SO**_**4**_ **at 115°C.** H_2_SO_4_ concentrations are **(a)** 0.2%, **(b)** 2.1%, or **(c)** 4.0% of H_2_SO_4_. Data were calculated from two independent experiments.

In order to find the concentrations of released products from SCB hydrolysis, the treatments were carried out at different H_2_SO_4_ concentrations ranging from 0.2 to 4.0% at 115°C for different hydrolysis times varying from 30 to 150 min. The concentrations of important components generated from the SCB solutions are shown in Figure 3. One can see that glucose and xylose are the main products, and the xylose concentration is always much higher than the glucose concentration because of the lower thermal stability of hemicellulose compared to that of cellulose [23,24]. The variation of arabinose and cellobiose in the range of H_2_SO_4_ concentration and hydrolysis time investigated was unremarkable. The concentrations of glucose and xylose increased with the extension of the reaction time. The highest glucose and xylose concentrations, 3.81 and 19.91 g/L, respectively, were observed at 150 min with 2.1% H_2_SO_4_ treatment. Under these conditions, 1.78 g/L arabinose, 1.53 g/L cellobiose, 3.58 g/L acetic acid, and 1.29 g/L phenolic compounds were achieved.

It needs to be stressed that an increase in acid concentration did not always result in an increase in glucose/xylose production. Generally speaking, increasing the acid concentration allows for a stronger reaction for breaking down the chemical bonds of cellulosic biomass, therefore yielding higher concentrations of hydrolyzed products [11]. However, further conversion of sugars to other substances, such as furfural and HMF, could potentially occur when excess acid is added [25]. The higher concentrations of furfural and HMF have been reported to damage microorganisms and inhibit cell growth and metabolism [22]. Our results showed the same trend; the sugar concentration was significantly decreased accompanied by an increase of furfural and HMF using 4.0% H_2_SO_4_. For the highest concentrations of furfural and HMF in the SCB hydrolysate below the lower limit of the toxic effect, no relevant data were presented in Figure 3.

For higher concentrations of sugars released, often with higher inhibitor generation, we define the hydrolysis efficiency (*E*) to inspect the optimum condition for the acid hydrolysis of SCB (Table 1). The *E* values increased from 1.59 to 3.35 when the reaction time changed from 30 min to 150 min at the lower H_2_SO_4_ concentration of 0.2% (*v/v*). Not surprisingly, an excess degree of acid hydrolysis, from a higher H_2_SO_4_ concentration or a longer acid hydrolysis time, led to a decrease of the*E* value. In terms of green chemistry and cost reduction, we prefer to keep the acid concentration as low as possible, and to shorten the reaction time as much as possible. Based on the above considerations, conditions of 2.1% (*v/v*) of H_2_SO_4_ for 90 min of hydrolysis were arguably the best, achieving the highest *E* ratio of 6.60 with a total amount of sugars of 24.88 g/L and an amount of inhibitors of 6.6 g/L.

**Table 1:** **Comparision of SCB hydrolyzed at different H**_**2**_ **SO**_**4**_ **concentrations for different reaction times**

**H**_**2**_ **SO**_**4**_ **(%,** ***v/v*** **)**	**Incubation time (min)**	**Total sugar** ^**a**^ **(g/L)**	**Total inhibitor** ^**b**^ **(g/L)**	**Efficiency** ^**c**^ **(** ***E*** **)**
0.2	30	5.66	2.55	1.59
	90	13.40	3.48	2.99
	150	16.32	3.87	3.35
2.1	30	9.96	2.58	2.78
	90	24.88	2.77	6.60
	150	27.30	4.87	4.60
4.0	30	11.99	3.44	2.70
	90	17.76	4.53	3.21
	150	18.89	5.43	2.94

^a^Total sugar = glucose + xylose + arabinose + cellobiose.

^b^Total inhibitor = acetic acid + phenol compounds.

^c^ *E* = total sugar/(1 + total inhibitor) [23].

### Optimization of hydrogen production from SCB hydrolysates

Our preliminary study revealed that *T. aotearoense* SCUT27/Δ*ldh* could grow and produce hydrogen in the SCB hydrolysate without any sterilization steps. Thus, in this study, all the biohydrogen production from SCB hydrolysates by SCUT27/Δ*ldh* was performed under non-sterilized anaerobic fermentation, which simplified the pretreatment process greatly. In order to identify if the cultured organisms after non-sterilized fermentation were still the strain *T. aotearoense* SCUT27/Δ*ldh*, the 16S rDNA gene was amplified, using the genomic DNA prepared from the fermentation broth as template, and then sequenced. The PCR products were cloned into the pMD™18-T vector and then transformed into *Escherichia coli* DH5α competent cells. Five single colonies randomly selected were isolated, and the 16S rDNA gene was sequenced. Alignment results showed more than 99% similarity in gene sequence, which confirmed that the screened samples were the targeted microorganisms (see Additional file 1).

Table 2 shows the level and range of two parameters investigated, the concentration of sulfuric acid and the treatment time. All parameters were taken at a central coded value considered as zero and studied at three different levels (-1, 0, and +1). In this case, a three-level factorial design resulting in a total number of 13 experiments was employed to fit the second-order polynomial model according to a design by Design-Expert 8.0. The statistical combinations of the critical parameters along with the maximum observed and predicted hydrogen production are also listed in Table 2. These predicted values were close to the observed ones in all sets of experiments. A highest hydrogen output of 149.54 mL and a lowest oneof 28.05 mL were observed. Two regression equations, Equation 1a for coded values and Equation 1b for actual experimental values, which are analogous to Equation 3, showed the hydrogen (*Y*) as a function of the test variables *X*_1_ (H_2_SO_4_ concentration) and *X*_2_ (treatment time):

**Table 2:** **Three-level factorial experimental design with experimental and predicted values using different concentrations of H**_**2**_ **SO**_**4**_ **and treatment times** ^**a**^

**Std**	**Type** ^**b**^	**Concentration (%)**		**Time (min)**		**Hydrogen (mL)**	
		**Code**	***X***_**1**_	**Code**	***X***_**2**_	**Experimental**	**Predicted**
1	F	−1	0.2	-1	30	28.05	14.38
2	CE	0	2.1	-1	30	46.90	72.67
3	F	1	4.0	-1	30	60.95	48.85
4	CE	−1	0.2	0	90	66.45	82.98
5	C	0	2.1	0	90	141.05	135.15
6	CE	1	4.0	0	90	91.80	105.20
7	F	−1	0.2	1	150	86.05	83.18
8	CE	0	2.1	1	150	125.05	129.22
9	F	1	4.0	1	150	94.45	93.15
10	C	0	2.1	0	90	145.21	135.15
11	C	0	2.1	0	90	149.54	135.15
12	C	0	2.1	0	90	138.09	135.15
13	C	0	2.1	0	90	131.78	135.15

^a^Design part was derived from the software Design-Expert 8.0.

^b^F = Factorial, CE = CentEdge, C = Center.

1a $$ {Y}_{\mathrm{coded}}=135.15 + 11.11{X}_1+28.27{X}_2-6.12{X}_1{X}_2-41.05{X_1}^2-34.20{X_2}^2 $$

1b $$ {Y}_{\mathrm{actual}}=-56.80+58.44{X}_1+2.29{X}_2-0.05{X}_1{X}_2-11.37{X_1}^2-9.50*{10}^{-3}{X_2}^2 $$

Where *Y* is the hydrogen production from SCB hydrolysates expressed in microliters (mL).

A statistical analysis, such as analysis of variance (ANOVA), is essential to test the significance and adequacy of the model. The ANOVA of the quadratic regression model demonstrated that the model is highly significant, evidenced by an *F*-value equal to 13.97 in the Fisher *F*-test and a very low probability value (*P*-value =0.0016) (Table 3) [26].

**Table 3:** **ANOVA for hydrogen production by** ***T. aotearoense*** **SCUT27/**Δ***ldh*** **with SCB hydrolysates as substrate** ^**a**^

**Factors**	**Sum of squares**	**Degrees of freedom**	**Mean square**	***F*** **-value**	***P*** **-value**	
Model	18368.05	5	3673.61	13.97	0.0016	significant
*X*_1_	740.37	1	740.37	2.82	0.1373	
*X*_2_	4796.85	1	4796.85	18.24	0.0037	
*X*_1_ *X*_2_	150.06	1	150.06	0.57	0.4747	
*X*_1_^2^	4654.76	1	4654.76	17.70	0.0040	
*X*_2_^2^	3230.99	1	3230.99	12.29	0.0099	
Residual	1840.87	7	262.98			
Lack of fit	1656.83	3	552.28	12.00	0.0181	significant
Pure error	184.04	4	46.01			
Cor total	20208.92	12				

^a^Coefficient of determination (*R* ^2^) = 0.9089. A model with an *F*-value of 13.97 implies that the model is significant. There is only a 0.16% chance that a model *F*-value this large could occur due to noise. Values of “Prob>*F*” less than 0.0500 indicate that model terms are significant. In this case B, A2, B2 are significant model terms. The “Lack of fit *F*-value” of 12.00 implies that the lack of fit is significant. There is only a 1.81% chance that a lack of fit *F*-value this large could occur due to noise. The “Pred R-Squared” of 0.3684 is not as close to the “Adj R-Squared” of 0.8438 as one might normally expect. This may indicate a large block effect or a possible problem with a model and/or data. Things to consider are model reduction, response transformation, and outliers, among others. “Adeq Precision” measures the signal-to-noise ratio. A ratio greater than 4 is desirable. A ratio of 10.962 indicates an adequate signal. This model can be used to navigate the design space.

**Figure 4:**
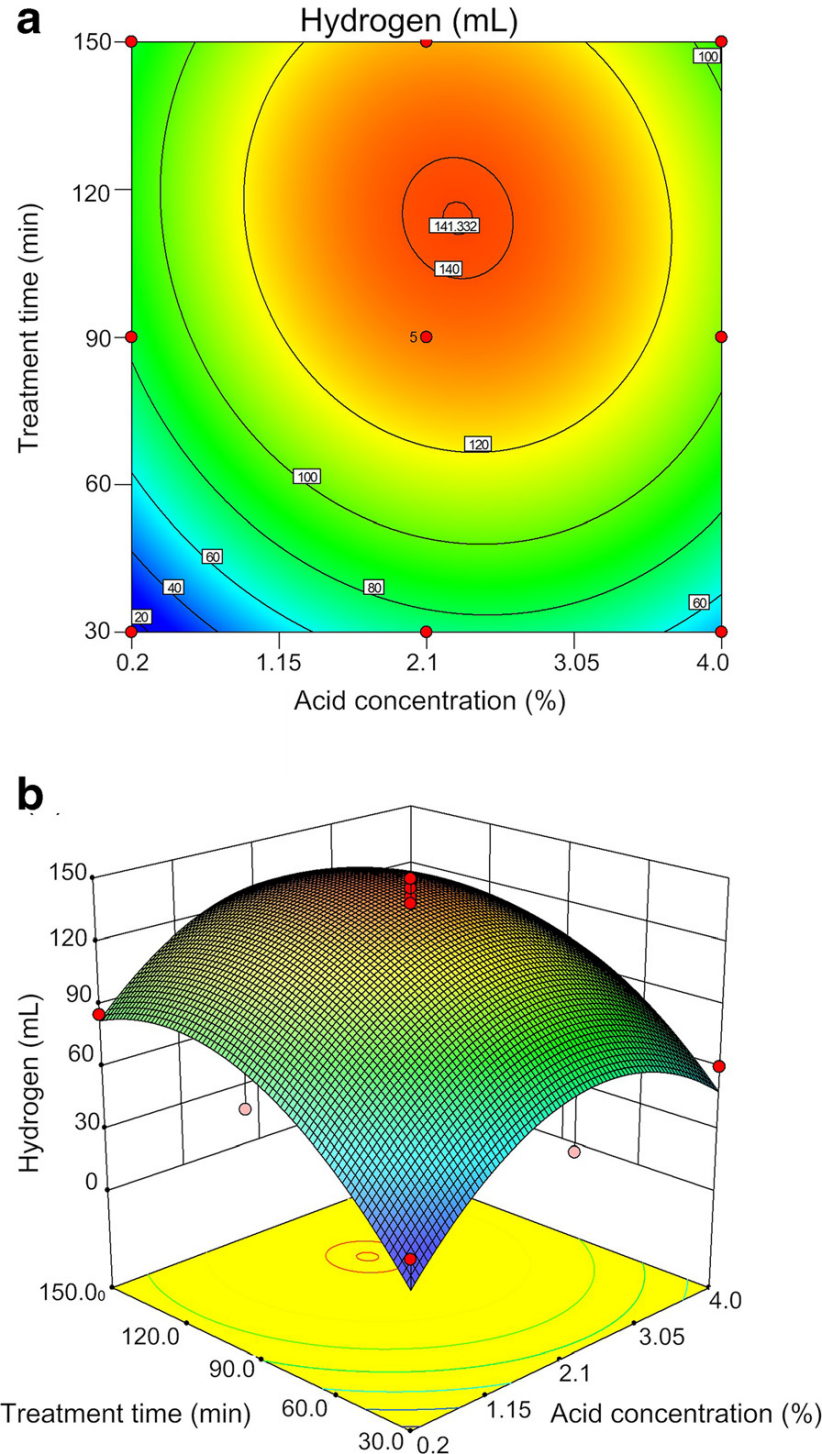
**Different plots of quadratic model of the effects of acid concentration and reaction time on the H**_**2**_ **production.** **(a)** two-dimensional contour plot and **(b)** three-dimensional diagram. The predicted optimum hydrogen production using sugarcane bagasse as the substrate was found at 2.3% H_2_SO_4_ and 114.2 min.

The two- and three-dimensional contour plots of the variation of hydrogen production with H_2_SO_4_ concentration and treatment time (Figure 4) are elliptical and have clear elongated diagonals, indicating significant interactive effects on hydrogen production (*Y*) between the two independent variables. Figure 4b has clear peaks, and the corresponding contour plot has clear maxima, indicating that maximum hydrogen could be achieved inside the design boundaries. The results depicted that the predicted 141.43 mL of maximum hydrogen production using SCB as substrate was found at 2.3% of H_2_SO_4_ and a treatment time of 114.2 min. Validation experiments (carried out in triplicate) were conducted to confirm the predicted optimal conditions, and gave a mean hydrogen production of 143.51 ± 2.29 mL H_2_, very close to the predicted value. The results suggested a strong correlation between cumulative hydrogen produced and *E* value, implying the importance of the relative amount of inhibitor to sugar concentration in hydrogen production.

Residual plots of the model were randomly distributed without any trends (not shown), validating the quadratic models.

### Hydrogen production from sugarcane bagasse hydrolysate

**Figure 5:**
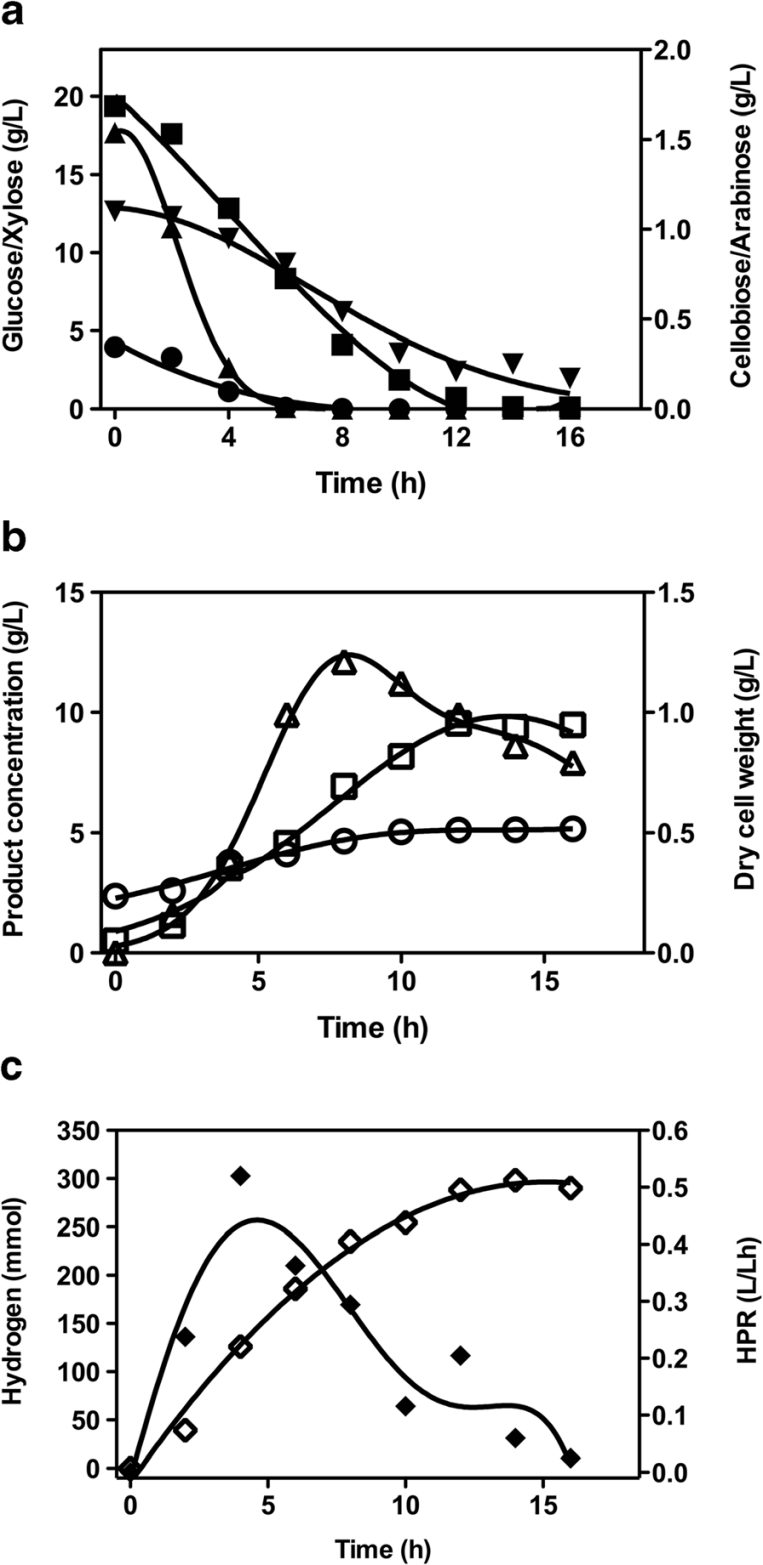
**Batch culture profiles of** ***T. aotearoense*** **SCUT27/**Δ***ldh*** **grown on the hydrolysate of sugarcane bagasse. (a)** Sugars consumption: glucose (circles), xylose (squares), cellobiose (up-facing triangles), arabinose (down-facing triangles); **(b)** dry cell weight (triangles), ethanol (squares), acetic acid (circles); and **(c)** hydrogen (open diamonds) and hydrogen production rate (solid diamonds).

The batch culture profiles clearly showed that *T. aotearoense* SCUT27/Δ*ldh* could grow and produce hydrogen effectively in a 5-L fermentor containing 2 L non-sterilized SCB hydrolysate. Glucose and cellobiose were depleted at 6 h. Xylose was almost completely utilized after 16 h cultivation, while arabinose was slowly consumed during the fermentation process (Figure 5a).

As shown in Figure 5b, the concentrations of the main liquid products, ethanol and acetic acid, increase with time and level off after 12 h incubation. The highest ethanol concentration reached 9.54 g/L with a yield of 0.42 g/g total sugars. The DCW climbed to 1.16 g/L at 8 h fermentation and then slowly declined. A small amount of acetic acid was produced at a maximum concentration of 5.12 g/L at the end of fermentation; however 2.37 g/L acetic acid originated from the acid pretreatment of SCB.

*T. aotearoense* SCUT27/Δ*ldh* can produce hydrogen efficiently using SCB hydrolysate (Figure 5c and Table 4). The final amount of hydrogen reached a value of 298.4 mmol. An average hydrogen molar yield of 1.86 mol H_2_/mol total sugar was obtained at the late fermentation period by this engineered strain. The achieved hydrogen yield was in the average range of previous reports (Table 4). Moreover, the HPR values increased over time in the initial stage of fermentation and decreased after the maximum HPR of 0.52 L/L · h obtained at the point of 4 h fermentation. Most thermophiles are able to hydrolyze various polysaccharides and ferment the released hexoses and pentoses to H_2_ with yields close to the theoretical maximum of 4 mol H_2_/mol hexose [6]. Niel*et al.* [27] reported a hydrogen yield of 3.33 mol/mol hexose using either *Caldicellulosiruptor saccharolyticus* on sucrose (70°C) or *Thermotogaelfii* on glucose (65°C), and similar yields were achieved by Mars et al. using hydrolyzed potato steam peels as the substrate [28]. Even so, ithas been pointed out that a drawback of thermophiles is that the HPR is relatively low, generally ranging from 0.01 to 0.2 L/L · h. However, the maximum HPR achieved by SCUT27/Δ*ldh* was much higher than those previously reported (Table 4). The performance of hydrogen production by SCUT27/Δ*ldh* using SCB hydrolysates in this study revealed a promising biohydrogen production process from cellulosic biomass.

**Table 4:** **Comparison of hydrogen production using various types of low-cost materials as substrate**

**Microorganism**	**Cultivation method**	**Temperature**	**Substrate**	**H**_**2**_ **yield (mol H**_**2**_ **/mol hexose)**	**HPR (L/L·h)**	**Ref.**
*Clostridium paraputrificum* M-21	Batch	45	Corn fiber	1.1	-	[29]
*C. bifermentans*	Batch	35	Wastewater sludge	2.1^a^	-	[30]
*Caldicellulosiruptor saccharolyticus*	Batch	70	Hydrolyzed potato steam peels	3.4	0.26^d^	[28]
*C. saccharolyticus*	Batch	70	Paper sludge hydrolysate	3.84	0.12	[31]
*Thermotoga neapolitana*	Batch	80	Hydrolyzed potato steam peels	3.3	0.20^d^	[28]
*Klebsiella oxytoca* HP1	Continuous	38	Bagasse	1.60^b^	0.35	[32]
*Clostridium butyricum* (immobilized)	Batch	37	Sugarcane juice	1.52	0.14^d^	[33]
NA	Batch	60	Cow manure	10.25^c^	0.02	[34]
Seed sludge	Batch	35	Pineapple waste	1.83	0.08^e^	[35]
Seed sludge	Batch	30-32	Sweet sorghum syrup	2.22	0.05	[4]
*C. butyricum*	Batch	37	SCB hydrolysate	1.73	0.07	[11]
*Thermoanaerobacterium thermosaccharolyticum* W16	Batch	60	Corn stover	-	0.25^d^	[36]
*T. aotearoense* SCUT27/Δ*ldh*	Batch	55	SCB hydrolysate	1.86	0.52	This study

^a^mmol H_2_/g COD.

^b^mmol H_2_/g solid.

^c^mL H_2_/g volatile solid.

^d^Obtained by calculation from reported data.

^e^L H_2_/g volatile solid/h.

It is important to note that all the sugar utilization (xylose, cellobiose, and arabinose) in the cultivation was started with glucose consumption at the initial stage, indicating that carbon catabolite repression (CCR) was not obvious for the strain. The rapid consumption of xylose with glucose might be the main reason for the high HPR by SCUT27/Δ*ldh* using SCB hydrolysates as substrate.

CCR is a tenacious bottleneck in the microbial production of bio-based chemicals from lignocellulose-derived sugar mixtures. A preferential sugar uptake (for example, glucose), accompanied by the blocking of less preferred sugars, leads to one of the major barriers in increasing the yield and productivity of the fermentation process [37]. Hence, the discovery of a strain with the capacity to co-utilize all of the sugars derived from biomass is one of the main tasks in cellulosic energy production [38]. Although several genetic and evolutionary engineering approaches achieved efficient pentose utilization in some industrial cell factories, such as those using *Zymomonas mobilis* [39] and *Saccharomyces cerevisiae* [40], CCR still remains a major bottleneck. However, it is encouraging that SCUT27/Δ*ldh* could consume hexose and pentose almost simultaneously in the SCB hydrolysate, as this could be advantageous in improving productivity and shortening fermentation time in lignocellulosic fuel production. In particular, the cellobiose utilizing capability of SCUT27/Δ*ldh* would help to reduce the need for additional saccharifying enzymes used in the hydrolysis of lignocellulose [19,41].

## Conclusions

This study demonstrated that a sulfuric acid hydrolysate of SCB was suitable for producing hydrogen by *T. aotearoense* SCUT27/Δ*ldh* due to the main compounds of xylose and glucose and low concentrations of inhibitors. The variations in acid concentration and treatment time affected the hydrolysis efficiency and the hydrogen production. The optimum conditions were found to be 2.3% H_2_SO_4_ and 114.2 min reaction time at 115°C. Research with larger batches in 5-L fermentation tanks finally produced 298.40 mmol hydrogen with an average molar yield of 1.86 mol H_2_/mol total sugar and a maximum HPR of 0.52 L/L · h, respectively. Also, there was no obvious CCR, which would be beneficial for higher hydrogen production and shorter retention time. All the thermophilic hydrogen performance results using non-sterilized SCB hydrolysates as substrate showed a favorable comparison with the results reported in the literature for sterilized fermentation. In particular, the higher HPR might give a more competitive edge for a process using inexpensive raw materials. Considering the low cost of SCB, the relatively moderate operation conditions, and the fact that there is no need for sterilization, hydrogen production by SCUT27/Δ*ldh* from the dilute acid treatment of SCB might be practically and economically attractive for industrial mass production.

## Methods

### Microorganism

The engineered strain of *T. aotearoense* SCUT27/Δ*ldh* was obtained by our group in a previous work [18]. Single colonies were selected and cultured to the exponential phase and subsequently maintained in 10-mL crimp-sealed anaerobic tubes in 25% glycerol and 75% growth medium at -80°C for long-term conservation. The cultures recovered from glycerol stocks were activated by transferring 2 mL of the stock culture into 4 mL of fresh modified MTC medium [18]. The serum tube was flushed with nitrogen to create anaerobic conditions and cultured at 55°C for about 12 h to reach an optical density (OD_600_) of 0.8. Then the cells were further enriched by inoculating 10% *v/v* of the previous culture into 12 mL fresh MTC medium and incubated at the given conditions to an OD_600_ of 1.0 prior to inoculum.

### SCB pretreatment

The SCB used in this study was obtained locally from the Guangzhou Sugarcane Industry Research Institute (Guangzhou, China). The SCB was air dried until the weight was constant. Then it was milled, screened through a 0.3-mm sieve, homogenized in a single lot, and kept at 4°C until use. The SCB consists of (*w/v*) glucan, 39.50 ± 0.66%; xylan, 19.77 ± 0.03%; araban, 2.02 ± 0.25%; klason lignin 21.04 ± 0.01%; acid-soluble lignin, 4.89 ± 0.21%; ash, 5.69 ± 0.01%; moisture, 6.85 ± 0.01% and other components.

### Acid hydrolysis

According to procedures for the acid hydrolysis of SCB [10,23], the dried SCB was hydrolyzed by 0.2%, 2.1%, and 4.0% (*v/v*) of sulfuric acid in an autoclave at 115°C. The time of the hydrolysis was controlled at 30, 90, and 150 min. For all conditions we used a liquid/solid ratio (LSR) of 15 mL liquid/g dry weight of SCB (modified from [11]). The solution was filtered through Whatman® filter papers, and the filtrate was adjusted to neutral using solid calcium hydroxide, followed by a centrifugation at 10,000 rpm for 20 min (Thermo Scientific Sorvall Legend RT Plus). The supernatant was adjusted to a pH of 6.8 with concentrated hydrochloric acid. Then the samples were analyzed for sugars and inhibitors by high performance liquid chromatography (HPLC). The hydrolysates from the SCB were added with the essential components of the buffer system, the nitrogen source, inorganic salt, and trace elements in the MTC medium recipes [18] and used as substrates to produce hydrogen by SCUT27/Δ*ldh*.

### Optimization of acid hydrolysis for hydrogen production

A response surface methodology (RSM) with a three-level factorial design (miscellaneous) was used as the experimental design model to optimize the key process parameters for enhanced hydrogen production. For two factors, the miscellaneous design offers some advantages, asit requires fewer experimental runs and allows efficient estimation ofquadratic surfaces, which usually works well for the optimization of the response within the region of the observation space [42,43]. For statistical calculations, the variables *X*_*i*_ (the uncoded value of the *i*th independent variable) were coded as *x*_*i*_ (the coded value of the *i*th independent variable) according to the following equation:

2 $$ {x}_i=\frac{X_i-{X}_i^{*}}{\Delta {X}_i} $$

where${X}_i^{*}$ is the value of *X*_*i*_ at the center point, and ∆*X*_*i*_ is the step change value.

In the present study, the levels of the variables and the experimental design (according to Design-Expert 8.0) are shown in Table 2. The hydrogen production amounts in volume were associated with simultaneous changes in sulfuric acid concentration (0.2, 2.1, and 4.0%) and the hydrolysis time (30, 90, and 150 min) of SCB. Accordingly, 13 experiments determined with the miscellaneous design were carried out for building quadratic models, with four replications of the center points to estimate experimental errors. The experimental data obtained from the miscellaneous design model experiments were represented in the following equation to predict the optimal conditions:

3 $$ Y={b}_0+{\displaystyle {\sum}_{i=1}^n}{b}_i{X}_i+{\displaystyle {\sum}_{i=1}^n}{b}_{i i}{X}_i^2+{\displaystyle {\sum}_{i=1}^{n-1}{\displaystyle {\sum}_{j= i+1}^n}}{b}_{i j}{X}_i{X}_j+{e}_i $$

where *X*_*i*_ are the input variables, which influence the response variable *Y*, *b*_0_ is the offset term, *b*_*i*_, *b*_*ii*_, and *b*_*ij*_ are the first-order, quadratic, and interaction coefficients, respectively, *n* is the number of factors, *i* and *j* are the index numbers for the factors, and *e*_*i*_ is the residual error [44,45].

Design-Expert 8.0 was used to analyze the experimental results and build the regression model, which helped us to predict the optimal processing parameters.

### Fermentation experiments

To evaluate the effect of the carbon source on the hydrogen production, cells were cultivated using different single carbon sources or a sugar mixture at a concentration of 5 g/L in 125-mL serum bottles at 55°C for 24 h or 48 h. The above-mentioned sugars included glucose, mannose, xylose, cellobiose, fructose, galactose, maltose, arabinose, lactose, sucrose, dextran, and beechwood xylan (xylooligosaccharide).

For the optimization study, the biohydrogen production was measured in 125-mL serum bottles containing 50 mL of acid-hydrolyzed SCB derived under different operating conditions. The contents were used directly without sterilization and inoculated with a seed culture of *T. aotearoense* SCUT27/Δ*ldh* in the late log phase of growth. The evolved gas was collected and analyzed by gas chromatography.

Batch reactor studies were carried out in a 5-L Biostat B fermentor (B. Braun, Germany) containing 2 L of non-sterilized SCB hydrolysate. The seeds of SCUT27/Δ*ldh* were inoculated into the fermentor with a ratio of 10% (*v/v*) and then cultured at 55°C for 16 h with a stirring rate of 100 rpm. The pH of the culture was kept at 6.5 by automatic addition of 2.5 mol/L NaOH. The liquid products were sampled at specified intervals to analyze the reducing sugars, ethanol, and organic acids by HPLC.

### Analytical methods

The hydrolysate was filtered through a 0.45-μm cellulose acetate membrane and analyzed by HPLC (Waters 2695, Milford, MA) for glucose, xylose, cellobiose, arabinose, acetic acid, and furfural. The culture broth after fermentation was neutralized with calcium carbonate and also filtered through a 0.45-μm filter for further analysis.

The reducing sugars, ethanol, and organic acids of the hydrolysates and the fermentation broth were analyzed by HPLC using an Aminex HPX-87P column (Bio-Rad, Hercules, CA), with 1 mmol/L H_2_SO_4_ as the mobile phase at a flow rate of 0.6 mL/min, and a refractive index detector [46]. The concentration of total phenolics in the hydrolysate was determined using a modified Folin-Ciocalteu method [47], with gallic acid (GA) as the standard. 500 μL of the sample solution was mixed with 500 μL of 1 N Folin-Ciocalteu reagent, and 1 mL of 20% Na_2_CO_3_ was added. After 10 min incubation at room temperature, the absorbance of the supernatant was read at 730 nm and compared to a standard curve of prepared GA solutions and expressed in terms of GA equivalents (grams of GA per liter).

The gas phase species from the 5-L fermentor were collected in a 30-L aluminum foil gasbag (Hua Rui Bo Yuan, Beijing, China). The gas volume was determined by water displacement and the contents of hydrogen and carbon dioxide were determined using a gas chromatograph (GC, Fuli 9790, China) equipped with a thermal conductivity detector (TCD) and a flame ionization detector (FID) through a TDX-01 column and an AE electric insulating oil analysis column [18].

The bacterial dry cell weight (DCW) was determined by a linear correlation equation from the optical density at 600 nm [19].

The SCB hydrolysis efficiencies (*E*) of sulfuric acid were calculated using the following equation:

4 $$ \left[\mathrm{E}\right]=\frac{{\displaystyle \sum}\mathrm{S}}{1+{\displaystyle \sum}\mathrm{I}} $$

where Σ*S* is the sum of the concentrations of all sugars in the hydrolysate (glucose, xylose, cellobiose, and arabinose) and Σ*I* is the sum of the inhibitor concentrations in the hydrolysate (acetic acid and total phenolics).

## Additional file

Additional file 1:
**Sequence alignment of 16S rDNA.** SCUT27, *Thermoanaerobacterium aotearoense* SCUT27. Numbers 3, 4, 6, 9, and 13 are the clone numbers. Results show that the similarity of 16S rDNA gene sequences is >99%.

## Abbreviations

CCR: carbon catabolite repression
DCW: dry cell weight
GC: Gas chromatograph
HMF: 5-hydroxymethyl furfural
HPLC: High performance liquid chromatography
HPR: hydrogen production rate
LSR: liquid/solid ratio
RSM: response surface methodology
SCB: sugarcane bagasse
