# Supplementary material for: Optimization of key factors affecting hydrogen production from sugarcane bagasse by a thermophilic anaerobic pure culture
Source: Biotechnol Biofuels. 2014 Aug 20;7:119. doi: 10.1186/s13068-014-0119-5 (PMC4147175; doi:10.1186/s13068-014-0119-5)
Supplement: Additional file 1: — Sequence alignment of 16S rDNA. SCUT27, Thermoanaerobacterium aotearoense SCUT27. Numbers 3, 4, 6, 9, and 13 are the clone numbers. Results show that the similarity of 16S rDNA gene sequences is >99%. [file 13068_2014_119_MOESM1_ESM.pdf]

```
        ********************************************   *** ************************** *********************
SCUT27 -TAGCGGCGGACGGGTGAGTAACGCGTGGACAATCTACCCTGTAGACCGGGATAACACCTCGAAAGGGGTGCTAATACCGGATAATGTCAAGAAGTGGCA    99
     3 -TAGCGGCGGACGGGTGAGTAACGCGTGGACAATCTACCCTGTAGACCGGGATAACACCTCGAAAGGGGTGCTAATACTGGATAATGTCAAGAAGTGGCA    99
     4 -TAGCGGCGGACGGGTGAGTAACGCGTGGACAATCTACCCTGTAGACCGGGGTAACACCTCGAAAGGGGTGCTAATACTGGATAATGTCAAGAAGTGGCA    99
     9 -TAGCGGCGGACGGGTGAGTAACGCGTGGACAATCTACCCTGTAGACCGGGATAACACCTCGAAAGGGGTGCTAATACTGGATAATGTCAAGAAGTGGCA    99
     6 -TAGCGGCGGACGGGTGAGTAACGCGTGGACAATCTACCCTGTAGACCGGGATAACACCTCGAAAGGGGTGCTAATACTGGATAATGTCAAGAAGTGGCA    99
    13 -TAGCGGCGGACGGGTGAGTAACGCGTGGACAATCTACCCTGTAGTTTGGGATAACACCTCGAAAGGGGTGCTAATACCGGATAATGTCAAGAAGTGGCA    99
 ruler 1.......10........20........30........40........50........60........70........80........90.......100

       ****** *********************************************************************************************
SCUT27 TCACTTATTGAAGAAAGGAGAAATCCGCTATAGGATGAGTCCGCGTCCCATTAGCTAGTTGGCGGGTAAAAGCCCACCAAGGCGACGATGGGTAGCCGG   199
     3 TCACTTATTGAAGAAAGGAGAAATCCGCTATAGGATGAGTCCGCGTCCCATTAGCTAGTTGGCGGGTAAAAGCCCACCAAGGCGACGATGGGTAGCCGG   199
     4 TCACTTATTGAAGAAAGGAGAAATCCGCTATAGGATGAGTCCGCGTCCCATTAGCTAGTTGGCGGGTAAAAGCCCACCAAGGCGACGATGGGTAGCCGG   199
     9 TCACTTATTGAAGAAAGGAGAAATCCGCTATAGGATGAGTCCGCGTCCCATTAGCTAGTTGGCGGGTAAAAGCCCACCAAGGCGACGATGGGTAGCCGG   199
     6 TCACTTATTGAAGAAAGGAGAAATCCGCTATAGGATGAGTCCGCGTCCCATTAGCTAGTTGGCGGGTAAAAGCCCACCAAGGCGACGATGGGTAGCCGG   199
    13 TCACTTTTTGAAGAAAGGAGAAATCCGCTATAGGATGAGTCCGCGTCCCATTAGCTAGTTGGCGGGTAAAAGCCCACCAAGGCGACGATGGGTAGCCGG   199
 ruler .......110.......120.......130.......140.......150.......160.......170.......180.......190.......200

       ******************************************************************************* ********************
SCUT27 CCTGAGAGGGTGAACGGCCACACTGGAACTGAGACACGGTCCAGACTCCTACGGGAGGCAGCAGTGGGGAATATTGTGCAATGGGGGAAACCCTGACACA   299
     3 CCTGAGAGGGTGAACGGCCACACTGGAACTGAGACACGGTCCAGACTCCTACGGGAGGCAGCAGTGGGGAATATTGTGTAATGGGGGAAACCCTGACACA   299
     4 CCTGAGAGGGTGAACGGCCACACTGGAACTGAGACACGGTCCAGACTCCTACGGGAGGCAGCAGTGGGGAATATTGTGCAATGGGGGAAACCCTGACACA   299
     9 CCTGAGAGGGTGAACGGCCACACTGGAACTGAGACACGGTCCAGACTCCTACGGGAGGCAGCAGTGGGGAATATTGTGCAATGGGGGAAACCCTGACACA   299
     6 CCTGAGAGGGTGAACGGCCACACTGGAACTGAGACACGGTCCAGACTCCTACGGGAGGCAGCAGTGGGGAATATTGTGCAATGGGGGAAACCCTGACACA   299
    13 CCTGAGAGGGTGAACGGCCACACTGGAACTGAGACACGGTCCAGACTCCTACGGGAGGCAGCAGTGGGGAATATTGTGCAATGGGGGAAACCCTGACACA   299
 ruler .......210.......220.......230.......240.......250.......260.......270.......280.......290.......300
```

Additional file 1: Sequence alignment of 16S rDNA. SCUT27, Thermoanaerobacterium aotearoense SCUT27. The 3, 4, 6, 9, and 13 are the clone numbers. Results show that the similarity of 16S rDNA gene sequences is >99%.

```
       ****************************************************************************************************
SCUT27 GCGACGCCGCGTGAGCGAAGAAGGCCTTCGGGTCGTAAAGCTCAATAGTATGGGAAGATAATGACGGTACCATACGAAAGCCCCGGCTAACTACGTGCCA   399
     3 GCGACGCCGCGTGAGCGAAGAAGGCCTTCGGGTCGTAAAGCTCAATAGTATGGGAAGATAATGACGGTACCATACGAAAGCCCCGGCTAACTACGTGCCA   399
     4 GCGACGCCGCGTGAGCGAAGAAGGCCTTCGGGTCGTAAAGCTCAATAGTATGGGAAGATAATGACGGTACCATACGAAAGCCCCGGCTAACTACGTGCCA   399
     9 GCGACGCCGCGTGAGCGAAGAAGGCCTTCGGGTCGTAAAGCTCAATAGTATGGGAAGATAATGACGGTACCATACGAAAGCCCCGGCTAACTACGTGCCA   399
     6 GCGACGCCGCGTGAGCGAAGAAGGCCTTCGGGTCGTAAAGCTCAATAGTATGGGAAGATAATGACGGTACCATACGAAAGCCCCGGCTAACTACGTGCCA   399
    13 GCGACGCCGCGTGAGCGAAGAAGGCCTTCGGGTCGTAAAGCTCAATAGTATGGGAAGATAATGACGGTACCATACGAAAGCCCCGGCTAACTACGTGCCA   399
 ruler .......310.......320.......330.......340.......350.......360.......370.......380.......390.......400

       ****************************************************************************************************
SCUT27 GCAGCCGCGGTAATACGTAGGGGGCGAGCGTTGTCCGGAATTACTGGGCGTAAAGAGCACGTAGGCGGCTGTAAAAGTCAGATGTGAAAAACCTGGGCTC   499
     3 GCAGCCGCGGTAATACGTAGGGGGCGAGCGTTGTCCGGAATTACTGGGCGTAAAGAGCACGTAGGCGGCTGTAAAAGTCAGATGTGAAAAACCTGGGCTC   499
     4 GCAGCCGCGGTAATACGTAGGGGGCGAGCGTTGTCCGGAATTACTGGGCGTAAAGAGCACGTAGGCGGCTGTAAAAGTCAGATGTGAAAAACCTGGGCTC   499
     9 GCAGCCGCGGTAATACGTAGGGGGCGAGCGTTGTCCGGAATTACTGGGCGTAAAGAGCACGTAGGCGGCTGTAAAAGTCAGATGTGAAAAACCTGGGCTC   499
     6 GCAGCCGCGGTAATACGTAGGGGGCGAGCGTTGTCCGGAATTACTGGGCGTAAAGAGCACGTAGGCGGCTGTAAAAGTCAGATGTGAAAAACCTGGGCTC   499
    13 GCAGCCGCGGTAATACGTAGGGGGCGAGCGTTGTCCGGAATTACTGGGCGTAAAGAGCACGTAGGCGGCTGTAAAAGTCAGATGTGAAAAACCTGGGCTC   499
 ruler .......410.......420.......430.......440.......450.......460.......470.......480.......490.......500

       ****************************************************************************************************
SCUT27 AACCGAGGGTGTGCATCTGAAACTATATAGCTTGAGTCAAGGAGAGGAGAGCGGAATTCCTGGTGTAGCGGTGAAATGCGTAGAGATCAGGAAGAATACC   599
     3 AACCGAGGGTGTGCATCTGAAACTATATAGCTTGAGTCAAGGAGAGGAGAGCGGAATTCCTGGTGTAGCGGTGAAATGCGTAGAGATCAGGAAGAATACC   599
     4 AACCGAGGGTGTGCATCTGAAACTATATAGCTTGAGTCAAGGAGAGGAGAGCGGAATTCCTGGTGTAGCGGTGAAATGCGTAGAGATCAGGAAGAATACC   599
     9 AACCGAGGGTGTGCATCTGAAACTATATAGCTTGAGTCAAGGAGAGGAGAGCGGAATTCCTGGTGTAGCGGTGAAATGCGTAGAGATCAGGAAGAATACC   599
     6 AACCGAGGGTGTGCATCTGAAACTATATAGCTTGAGTCAAGGAGAGGAGAGCGGAATTCCTGGTGTAGCGGTGAAATGCGTAGAGATCAGGAAGAATACC   599
    13 AACCGAGGGTGTGCATCTGAAACTATATAGCTTGAGTCAAGGAGAGGAGAGCGGAATTCCTGGTGTAGCGGTGAAATGCGTAGAGATCAGGAAGAATACC   599
 ruler .......510.......520.......530.......540.......550.......560.......570.......580.......590.......600
```

```
       ***** **********************************************************************************************
SCUT27 AGTGGCGAAAGCGGCTCTCTGGACTTGAACTGACGCTGAGGTGCGAAAGCGTGGGGAGCAAACAGGATTAGATACCCTGGTAGTCCACGCCGTAAACGAT   699
     3 AGTGGCGAAAGCGGCTCTCTGGACTTGAACTGACGCTGAGGTGCGAAAGCGTGGGGAGCAAACAGGATTAGATACCCTGGTAGTCCACGCCGTAAACGAT   699
     4 AGTGGCGAAAGCGGCTCTCTGGACTTGAACTGACGCTGAGGTGCGAAAGCGTGGGGAGCAAACAGGATTAGATACCCTGGTAGTCCACGCCGTAAACGAT   699
     9 AGTGGCGAAAGCGGCTCTCTGGACTTGAACTGACGCTGAGGTGCGAAAGCGTGGGGAGCAAACAGGATTAGATACCCTGGTAGTCCACGCCGTAAACGAT   699
     6 AGTGGCGAAAGCGGCTCTCTGGACTTGAACTGACGCTGAGGTGCGAAAGCGTGGGGAGCAAACAGGATTAGATACCCTGGTAGTCCACGCCGTAAACGAT   699
    13 AGTGGTGAAAGCGGCTCTCTGGACTTGAACTGACGCTGAGGTGCGAAAGCGTGGGGAGCAAACAGGATTAGATACCCTGGTAGTCCACGCCGTAAACGAT   699
 ruler ......610.......620.......630.......640.......650.......660.......670.......680.......690.......700

       ****************************************************************************************************
SCUT27 GGATACTAGGTGTGGGTGAGGAATCATCCGTGCCGGAGTTAACGCAATAAGTATCCCGCCTGGGGAGTACGGCCGCAAGGTTGAAACTCAAAGGAATTGA   799
     3 GGATACTAGGTGTGGGTGAGGAATCATCCGTGCCGGAGTTAACGCAATAAGTATCCCGCCTGGGGAGTACGGCCGCAAGGTTGAAACTCAAAGGAATTGA   799
     4 GGATACTAGGTGTGGGTGAGGAATCATCCGTGCCGGAGTTAACGCAATAAGTATCCCGCCTGGGGAGTACGGCCGCAAGGTTGAAACTCAAAGGAATTGA   799
     9 GGATACTAGGTGTGGGTGAGGAATCATCCGTGCCGGAGTTAACGCAATAAGTATCCCGCCTGGGGAGTACGGCCGCAAGGTTGAAACTCAAAGGAATTGA   799
     6 GGATACTAGGTGTGGGTGAGGAATCATCCGTGCCGGAGTTAACGCAATAAGTATCCCGCCTGGGGAGTACGGCCGCAAGGTTGAAACTCAAAGGAATTGA   799
    13 GGATACTAGGTGTGGGTGAGGAATCATCCGTGCCGGAGTTAACGCAATAAGTATCCCGCCTGGGGAGTACGGCCGCAAGGTTGAAACTCAAAGGAATTGA   799
 ruler .......710.......720.......730.......740.......750.......760.......770.......780.......790.......800

       ****************************************************************************************************
SCUT27 CGGGGGCCCGCACAAGCAGCGGAGCATGTGGTTTAATTCGAAGCAACGCGAAGAACCTTACCAGGGCTTGACATCCACAGAATCTGGTAGAAATACCGGA   899
     3 CGGGGGCCCGCACAAGCAGCGGAGCATGTGGTTTAATTCGAAGCAACGCGAAGAACCTTACCAGGGCTTGACATCCACAGAATCTGGTAGAAATACCGGA   899
     4 CGGGGGCCCGCACAAGCAGCGGAGCATGTGGTTTAATTCGAAGCAACGCGAAGAACCTTACCAGGGCTTGACATCCACAGAATCTGGTAGAAATACCGGA   899
     9 CGGGGGCCCGCACAAGCAGCGGAGCATGTGGTTTAATTCGAAGCAACGCGAAGAACCTTACCAGGGCTTGACATCCACAGAATCTGGTAGAAATACCGGA   899
     6 CGGGGGCCCGCACAAGCAGCGGAGCATGTGGTTTAATTCGAAGCAACGCGAAGAACCTTACCAGGGCTTGACATCCACAGAATCTGGTAGAAATACCGGA   899
    13 CGGGGGCCCGCACAAGCAGCGGAGCATGTGGTTTAATTCGAAGCAACGCGAAGAACCTTACCAGGGCTTGACATCCACAGAATCTGGTAGAAATACCGGA   899
 ruler .......810.......820.......830.......840.......850.......860.......870.......880.......890.......900
```

```
       ****************************************************************************************************
SCUT27 GTGCCTCGAAAGAGGAGCTGTGAGACAGGTGGTGCATGGTTGTCGTCAGCTCGTGTCGTGAGATGTTGGGTTAAGTCCCGCAACGAGCGCAACCCCTGTT   999
     3 GTGCCTCGAAAGAGGAGCTGTGAGACAGGTGGTGCATGGTTGTCGTCAGCTCGTGTCGTGAGATGTTGGGTTAAGTCCCGCAACGAGCGCAACCCCTGTT   999
     4 GTGCCTCGAAAGAGGAGCTGTGAGACAGGTGGTGCATGGTTGTCGTCAGCTCGTGTCGTGAGATGTTGGGTTAAGTCCCGCAACGAGCGCAACCCCTGTT   999
     9 GTGCCTCGAAAGAGGAGCTGTGAGACAGGTGGTGCATGGTTGTCGTCAGCTCGTGTCGTGAGATGTTGGGTTAAGTCCCGCAACGAGCGCAACCCCTGTT   999
     6 GTGCCTCGAAAGAGGAGCTGTGAGACAGGTGGTGCATGGTTGTCGTCAGCTCGTGTCGTGAGATGTTGGGTTAAGTCCCGCAACGAGCGCAACCCCTGTT   999
    13 GTGCCTCGAAAGAGGAGCTGTGAGACAGGTGGTGCATGGTTGTCGTCAGCTCGTGTCGTGAGATGTTGGGTTAAGTCCCGCAACGAGCGCAACCCCTGTT   999
 ruler .......910.......920.......930.......940.......950.......960.......970.......980.......990......1000

       ******************************************************* ********************************************
SCUT27 GGTAGTTACCAGCGTAAAGACGGGGACTCTACCGAGACTGCCGTGGATAACACGGAGGAAGGCGGGGATGACGTCAAATCATCATGCCCTTTATGCCCTG  1099
     3 GGTAGTTACCAGCGTAAAGACGGGGACTCTACCGAGACTGCCGTGGATAACACGGAGGAAGGCGGGGATGACGTCAAATCATCATGCCCTTTATGCCCTG  1099
     4 GGTAGTTACCAGCGTAAAGACGGGGACTCTACCGAGACTGCCGTGGATAACACGGAGGAAGGCGGGGATGACGTCAAATCATCATGCCCTTTATGCCCTG  1099
     9 GGTAGTTACCAGCGTAAAGACGGGGACTCTACCGAGACTGCCGTGGATAACACGGCGGAAGGCGGGGATGACGTCAAATCATCATGCCCTTTATGCCCTG  1099
     6 GGTAGTTACCAGCGTAAAGACGGGGACTCTACCGAGACTGCCGTGGATAACACGGAGGAAGGCGGGGATGACGTCAAATCATCATGCCCTTTATGCCCTG  1099
    13 GGTAGTTACCAGCGTAAAGACGGGGACTCTACCGAGACTGCCGTGGATAACACGGAGGAAGGCGGGGATGACGTCAAATCATCATGCCCTTTATGCCCTG  1099
 ruler ......1010......1020......1030......1040......1050......1060......1070......1080......1090......1100

       ****************************************************************************************************
SCUT27 GGCTACACACGTGCTACAATGGCCTGAACAGAGGGCAGCGAAGGAGCGATCCGGAGCGAATCCCAGAAAACAGGTCCCAGTTCAGATTGCAGGCTGCAAC  1199
     3 GGCTACACACGTGCTACAATGGCCTGAACAGAGGGCAGCGAAGGAGCGATCCGGAGCGAATCCCAGAAAACAGGTCCCAGTTCAGATTGCAGGCTGCAAC  1199
     4 GGCTACACACGTGCTACAATGGCCTGAACAGAGGGCAGCGAAGGAGCGATCCGGAGCGAATCCCAGAAAACAGGTCCCAGTTCAGATTGCAGGCTGCAAC  1199
     9 GGCTACACACGTGCTACAATGGCCTGAACAGAGGGCAGCGAAGGAGCGATCCGGAGCGAATCCCAGAAAACAGGTCCCAGTTCAGATTGCAGGCTGCAAC  1199
     6 GGCTACACACGTGCTACAATGGCCTGAACAGAGGGCAGCGAAGGAGCGATCCGGAGCGAATCCCAGAAAACAGGTCCCAGTTCAGATTGCAGGCTGCAAC  1199
    13 GGCTACACACGTGCTACAATGGCCTGAACAGAGGGCAGCGAAGGAGCGATCCGGAGCGAATCCCAGAAAACAGGTCCCAGTTCAGATTGCAGGCTGCAAC  1199
 ruler ......1110......1120......1130......1140......1150......1160......1170......1180......1190......1200
```

```
       ****************************************************************************************************
SCUT27 CCGCCTGCATGAAGACGGAGTTGCTAGTAATCGCGGATCAGCATGCCGCGGTGAATACGTTCCCGGGCCTTGTACACACCGCCCGTCACACCACGAGAGT 1299
     3 CCGCCTGCATGAAGACGGAGTTGCTAGTAATCGCGGATCAGCATGCCGCGGTGAATACGTTCCCGGGCCTTGTACACACCGCCCGTCACACCACGAGAGT 1299
     4 CCGCCTGCATGAAGACGGAGTTGCTAGTAATCGCGGATCAGCATGCCGCGGTGAATACGTTCCCGGGCCTTGTACACACCGCCCGTCACACCACGAGAGT 1299
     9 CCGCCTGCATGAAGACGGAGTTGCTAGTAATCGCGGATCAGCATGCCGCGGTGAATACGTTCCCGGGCCTTGTACACACCGCCCGTCACACCACGAGAGT 1299
     6 CCGCCTGCATGAAGACGGAGTTGCTAGTAATCGCGGATCAGCATGCCGCGGTGAATACGTTCCCGGGCCTTGTACACACCGCCCGTCACACCACGAGAGT 1299
    13 CCGCCTGCATGAAGACGGAGTTGCTAGTAATCGCGGATCAGCATGCCGCGGTGAATACGTTCCCGGGCCTTGTACACACCGCCCGTCACACCACGAGAGT 1299
 ruler ......1210......1220......1230......1240......1250......1260......1270......1280......1290......1300

       **************************************************
SCUT27 TTACAACACCCGAAGTCAGTGACCTAACCGTAAGGAAGGAGCTGCCGAAG 1349
     3 TTACAACACCCGAAGTCAGTGACCTAACCGTAAGGAAGGAGCTGCCGAAG 1349
     4 TTACAACACCCGAAGTCAGTGACCTAACCGTAAGGAAGGAGCTGCCGAAG 1349
     9 TTACAACACCCGAAGTCAGTGACCTAACCGTAAGGAAGGAGCTGCCGAAG 1349
     6 TTACAACACCCGAAGTCAGTGACCTAACCGTAAGGAAGGAGCTGCCGAAG 1349
    13 TTACAACACCCGAAGTCAGTGACCTAACCGTAAGGAAGGAGCTGCCGAAG 1349
 ruler ......1310......1320......1330......1340......1350
```
